# The kinectome: A comprehensive kinematic map of human motion in health and disease

**DOI:** 10.1111/nyas.14860

**Published:** 2022-07-15

**Authors:** Emahnuel Troisi Lopez, Pierpaolo Sorrentino, Marianna Liparoti, Roberta Minino, Arianna Polverino, Antonella Romano, Anna Carotenuto, Enrico Amico, Giuseppe Sorrentino

**Affiliations:** ^1^ Department of Motor Sciences and Wellness University of Naples “Parthenope” Naples Italy; ^2^ Institut de Neurosciences des Systèmes Aix‐Marseille Université Marseille France; ^3^ Department of Developmental and Social Psychology University “La Sapienza” of Rome Rome Italy; ^4^ Institute for Diagnosis and Treatment Hermitage Capodimonte Naples Italy; ^5^ Alzheimer Unit and Movement Disorders Clinic Department of Neurology Cardarelli Hospital Naples Italy; ^6^ Institute of Bioengineering, Center for Neuroprosthetics EPFL Geneva Switzerland; ^7^ Department of Radiology and Medical Informatics University of Geneva (UNIGE) Geneva Switzerland; ^8^ Institute of Applied Sciences and Intelligent Systems CNR Pozzuoli Italy

**Keywords:** gait analysis, movement pattern, network, Parkinson's disease

## Abstract

Human voluntary movement stems from the coordinated activations in space and time of many musculoskeletal segments. However, the current methodological approaches to study human movement are still limited to the evaluation of the synergies among a few body elements. Network science can be a useful approach to describe movement as a whole and to extract features that are relevant to understanding both its complex physiology and the pathophysiology of movement disorders. Here, we propose to represent human movement as a network (that we named the *kinectome*), where nodes represent body points, and edges are defined as the correlations of the accelerations between each pair of them. We applied this framework to healthy individuals and patients with Parkinson's disease, observing that the patients’ kinectomes display less symmetrical patterns as compared to healthy controls. Furthermore, we used the kinectomes to successfully identify both healthy and diseased subjects using short gait recordings. Finally, we highlighted topological features that predict the individual clinical impairment in patients. Our results define a novel approach to study human movement. While deceptively simple, this approach is well‐grounded, and represents a powerful tool that may be applied to a wide spectrum of frameworks.

## Graphical abstract

Here, we propose to represent human movement as a network (that we named kinectome), where nodes represent body points, and edges are defined as the correlations of the accelerations between each pair of them. We applied this framework in healthy individuals and patients with Parkinson's disease, observing that the patients’ kinectomes display less symmetrical patterns as compared to healthy controls. Our results define a novel approach to study human movement.

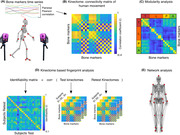

## INTRODUCTION

Movement is essential to human life and survival.^1^ As a consequence, movement impairment significantly reduces quality of life and individual autonomy. Thus, the study of the characteristics of the voluntary movement is of broad interest in multiple frameworks. However, an appropriate description of human movement requires taking into account multiple simultaneous interactions,^2^ stemming from the coordinated activations of several musculoskeletal segments,^3^, ^4^ and resulting in complex patterns.^5^ Such patterns are fine‐tuned, and also small changes can lead to physiologically relevant effects.^6^ Therefore, an accurate characterization of these patterns requires precise measurements and appropriate mathematical methods, in order to capture and describe their complex interactions.

To date, human movement kinematics, and notably gait kinematics, has been approached focusing on specific body segments, or conveying complex patterns into a few synthetic parameters.^7^, ^8^, ^9^, ^10^ While this approach is useful, it inevitably leads to loss of information, providing a summary picture of human movement rather than a comprehensive description of the complex patterns of interactions that generated it. Yet, whole‐body interactions are needed for a comprehensive account of movement dynamics.^11^, ^12^

Complex network theory is a methodological approach to integrate into a unique explanatory framework, complex systems consisting of a large number of interconnected elements.^13^ This approach allows the description of the properties of the network and, ultimately, of its functioning. The complex networks may be analyzed using a host of mathematical techniques, such as graph theory, a branch of mathematics dedicated to the study of the topological properties of the networks. Algebraically, graphs are represented as adjacency matrices, square arrays of numbers wherein rows and columns correspond to nodes and individual entries give the connection between each node and all the others.^14^ Network analysis, given its ability to capture the properties of the network as a whole, but also to analyze the contribution of each individual element to the organization of the entire network, finds wide applications in a large number of disciplines (e.g., physics, sociology, epidemiology, climatology, and neuroscience).^15^, ^16^, ^17^, ^18^

As a consequence, network science may lend itself nicely to describe the complex patterns generated by motor behaviors, and extract features that are relevant to the pathophysiology of movement disorders.^19^ Indeed, in the last decades, network science has been extensively applied to characterize the aspects of neurological disorders.^13^ Recently, the first applications of network analysis to the study of human movement proved successful. Utilizing electromyography, Boonstra *et al*. analyzed the network of the leg muscles, detecting the presence of lower and higher frequency components related to between and within legs connectivity, respectively.^20^ The authors suggested that network analysis may be suitable to study the motor system also in a clinical setting. In another study, Kerkman *et al*. investigated a combined musculoskeletal network structure. They examined the different frequency‐specific muscle networks during postural control.^21^ The study showed that the examined networks presented frequency‐specific relationships with the synaptic input to motor neurons.

Despite these first efforts, to date a comprehensive network description of the kinematics of movement is still lacking. To overcome this deficiency, borrowing concepts from network science, we set out to represent certain anatomical points as nodes, and their coaccelerations throughout gait as edges, thereby defining the *network of human movement*. With this approach, we aimed at identifying the large‐scale characteristics of the human gait. Hence, we considered the whole body as an integrated and synergistic system, whose individual musculoskeletal segments are in a constant and reciprocal biomechanical relationship constrained by the individual anatomical characteristics.

To this end, we utilized a three‐dimensional motion analysis stereophotogrammetric system, which is the gold standard for quantitative analysis of movement,^22^ and is widely applied for the assessment of motor skills in health and disease.^23^, ^24^, ^25^, ^26^, ^27^ Specifically, we captured the position of reflective markers applied on specific bone reference points during gait. Each bone marker was considered as a node, and the edges linking the nodes were defined by the covariance of the acceleration and jerk (i.e., the first derivative of acceleration with respect to time) between each pair of bone markers. We named the resulting network the human *kinectome*. We focused on acceleration and its derivative (i.e., jerk) for our analysis since those kinematic measures are mainly associated with smoothness of gait and quality of movement control.^28^, ^29^ Indeed, through acceleration and its tuning, we are able to properly control speed.

Then, we characterized the human kinectome in a cohort of healthy subjects (HS). Furthermore, in order to explore the clinical relevance of our framework, we compared the kinectomes of individuals affected by Parkinson's disease (PD), a neurodegenerative disorder which disrupts the motor patterns of the patient,^30^ to those of matched healthy controls (HC). Kinematics in PD patients has been widely investigated, with several studies focusing on different aspects of motor impairment, including variability, asymmetry, smoothness, and stability of gait.^31^, ^32^ Hence, PD kinematics emerges as a natural scope for the proposed approach. We hypothesized that PD patients would be less capable of maintaining an optimal motor strategy, as opposed to HC. According to this hypothesis, we first explored the structure of the kinectomes, expecting a dysregulated (i.e., more variable) organization in patients with respect to controls. Then, to test the reliability of the kinectome, we performed an identifiability analysis,^33^ identifying subjects based on their kinectomes, similarly to a *motion fingerprint*. This idea is in analogy to recent evidence showing that dysregulated activity would make brain network identifiability harder in patients as compared to healthy people.^34^ Finally, we hypothesized that our technique was able to capture clinically relevant features. To test this hypothesis, we extracted the nodal topological features of the kinectomes of the PD patients, and used them to predict the level of clinical impairment, as measured by the Unified Parkinson's Disease Rating Scale part III (UPDRS).^35^

## MATERIALS AND METHODS

### Participants

Sixty HS, including 38 males and 22 females, were recruited (mean age 58.7 ± 12.7 years). Exclusion criteria were the following: (1) Mini‐Mental State Examination < 24;^36^ (2) Frontal Assessment Battery < 12;^37^ (3) Beck Depression Inventory II > 13;^38^ neurological or psychiatric disorders; (4) intake of psychoactive drugs; and (5) physical or medical conditions causing motor impairment.

To test the validity of our methods in a clinical setting, we used the data of 23 patients (mean age 65.3 ± 11.6) affected by PD and 23 HC, matched for age, sex, and education. The subjects included in this study are partially overlapping with those included in a previous study.^39^ Parkinsonians were tested in off‐medicament state. Inclusion criteria were: (1) Hoehn and Yahr score ≤ 3 while off‐medicament;^40^ (2) disease duration < 10 years; and (3) antiparkinsonian treatment at a stable dosage. All participants signed an informed consent in accordance with the declaration of Helsinki. The study was approved by the “Azienda Ospedaliera di Rilievo Nazionale A. Cardarelli'” Ethic Committee (protocol number: 00019628).

### Stereophotogrammetric acquisition

The acquisitions were carried out in the Motion Analysis Laboratory of the University of Naples Parthenope. Gait data were recorded through a stereophotogrammetric system for motion analysis composed of eight infrared cameras (ProReflex Unit—Qualisys Inc., Gothenburg, Sweden), capturing (at 120 frame per second) the light reflected by 21 passive markers positioned on the naked skin of the participants. The markers were placed in correspondence of bone landmarks, based on a modified version of the Davis protocol.^41^ We asked the participants to walk in a straight path choosing their preferred walking speed. For each participant, two gait acquisitions were performed, each of which included one complete left and right gait cycle. A complete gait cycle is defined as starting with the heel touching the ground, and finishing with the next contact with the ground of the same heel. Through the Qualisys Track Manager software, we obtained the three‐dimensional position of each bone marker during the gait cycle. Hence, we could calculate the time series for acceleration and jerk (the first derivative of acceleration with respect to time) of each bone marker.

### Introducing the kinectome

**FIGURE 1:**
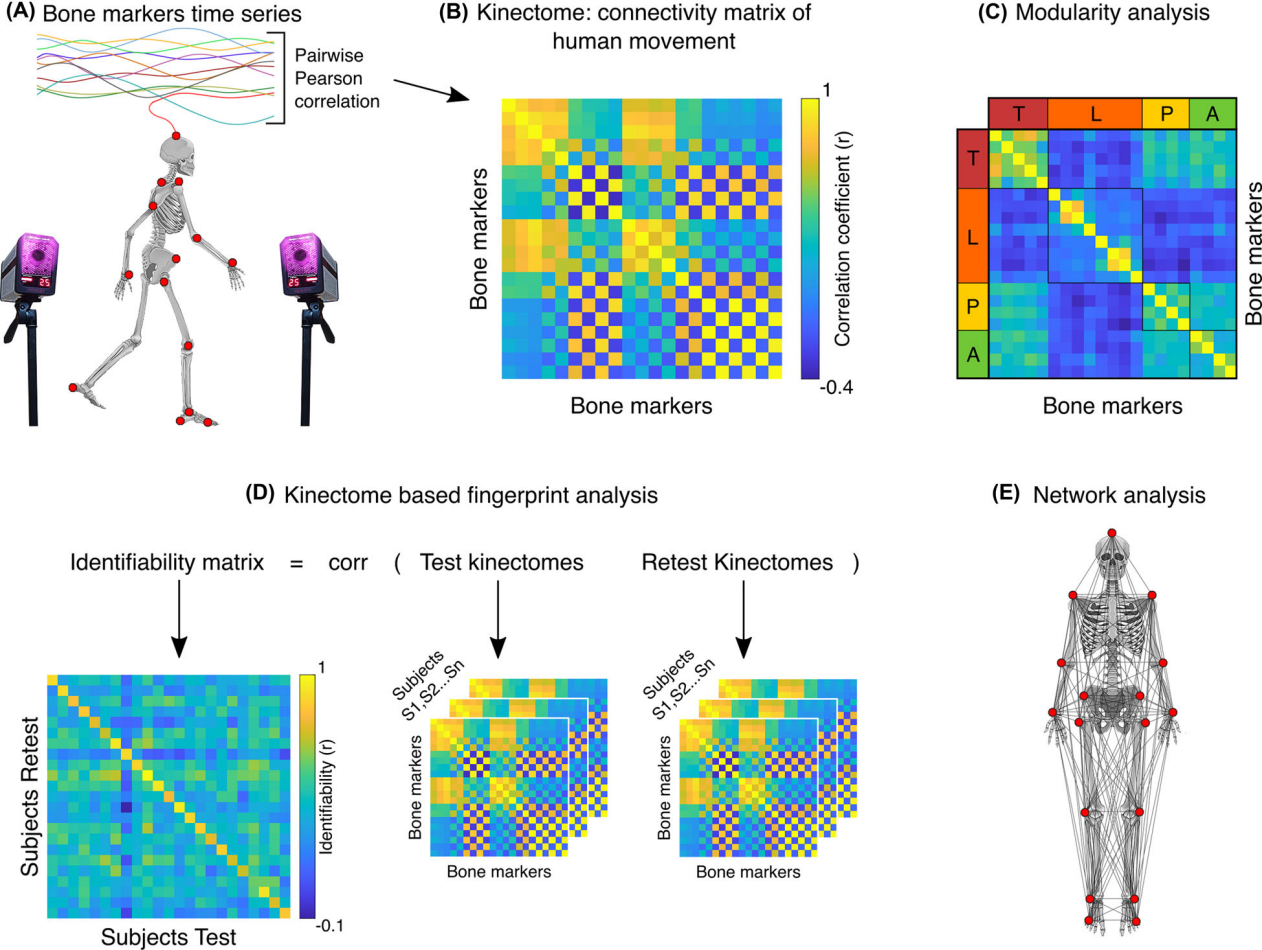
Scheme of kinectome analysis. (A) Marker positions of the bone landmarks. Acceleration and jerk time series are computed based on the positions of the markers during the gait cycle, as recorded by a stereophotogrammetric system. (B) Kinectome: the covariance matrix is computed correlating each pair of the bone markers acceleration or jerk time series; different kinectomes were built, based on the mediolateral and anteroposterior axis, and separately taking into account the accelerations and the jerks. (C) Functional network modularity was investigated using the Louvain method, an algorithm customarily employed for community detection. (D) Schematic illustration of the fingerprint analysis. Two kinectomes (named *test* and *retest*) have been computed for each subject. The identifiability matrix is obtained by correlating the test and retest kinectomes of each subject. The main diagonal displays self‐identifiability. (E) Graphical representation of the bone markers network used for the topological analysis. Note that the bone markers positioned on the back of the body are not visible.

We computed the Pearson's correlation coefficients between each pair of the time series representing the bone markers (see also Figure 1A,B), and defined the kinectome as the covariance matrix, which conveys whole‐body interactions in a pairwise fashion. Hence, using 21 markers as nodes, we obtained a symmetric matrix containing 420 edges (excluding the main diagonal elements which represent the correlation of a node with itself). Only 210 edges (since the kinectome is symmetric) were used in the subsequent analyses.

**FIGURE 2:**
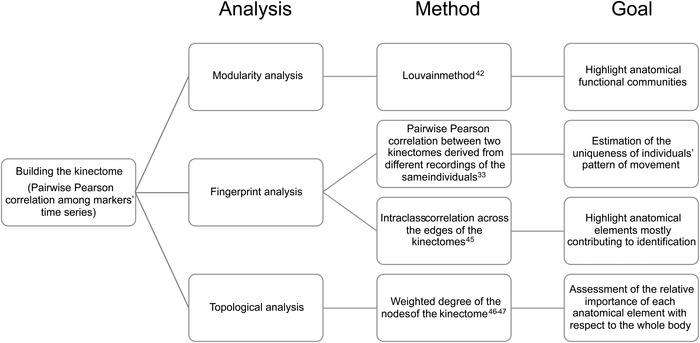
Schematic description of the network analysis. The flowchart describes the methodological approaches applied to the kinectomes. Three main network frameworks were explored: modularity,^42^ fingerprint,^33^, ^45^ and topology.^46^ For each of them, the methodological approach and the aim of the analysis have been highlighted.

Time series from acceleration and jerk of the 21 markers along the three axes of movement (i.e., mediolateral, anteroposterior, and vertical) were used to build six kinectomes for each subject (2 kinematic units × 3 axes of movement). First, we explored the kinectomes heterogeneity within and between groups (PD patients and controls), by comparing mean and standard deviations of the kinectomes. On the one hand, the analysis of the mean allows to understand the level of motor synchronization, and may help to understand whether a clinical condition is able to alter it. On the other hand, the analysis of the standard deviation allows to assess the variability of the motor patterns within a group, which in turn highlights anatomical elements affected by the disease. After those preliminary investigations, we then characterized the kinectomes utilizing a graph‐theoretical approach, as detailed in the next sections, and shown in the flowchart (Figure 2).

### Modularity analysis

Modularity measures the strength of division of a network into modules or communities. We assessed the community structure (i.e., partition) of each group‐averaged kinectome (anteroposterior, mediolateral, and vertical, separately), in both healthy and PD patients, by using the Louvain (with consensus clustering across 100 iterations) method for identifying communities in large networks (Figure 1C).^42^, ^43^ This is a method that detects communities by optimizing the modularity (Q) of the graph, defined as:

$$Q\;=\frac{1}{2m}\;\sum_{ij}\left(A_{ij}-\frac{k_{i}k_{j}}{2m}\right)\delta \left(c_{i},c_{j}\right),$$

where *m* is the sum of weights of all the edges in the network, $A_{ij}$ is the weight of the edge between *i* and *j*, $k_{i}$ and $k_{j}$ are the sum of the weights of the edges connected to nodes *i* and *j*, respectively, and $\delta (c_{i},c_{j})$ is the Kronecker delta between community $c_{i}\;\mathrm{and}\;c_{j}.$ ^42^

With this approach, we were able to determine which body elements were recognized as belonging to the same group, based on their acceleration and jerk motor patterns, creating an allegiance matrix.^44^ The aim was to identify, in a data‐driven fashion, functional dynamical clusters within healthy and diseased kinectomes during gait. The modularity analysis allows to divide the body into sets of anatomical elements working together toward a motor task. While symmetrical organization of these groups is expected in health, individuals with PD might show altered distributions, highlighting which are the most affected anatomical elements.

### Fingerprint analysis

Can we identify individuals based solely on their motion patterns, that is, their kinectomes? To address this question, we took inspiration from previous studies on fingerprint in human functional brain connectomes extracted from functional magnetic resonance imaging and magnetoencephalography data.^33^, ^34^ In a recent work,^33^ the authors defined a mathematical object known as identifiability matrix (IM), which encodes the information about the self‐similarity (I‐self, main diagonal elements) of each subject with herself/himself, comparing two recording sessions, and the similarity of each subject with the others. To build an IM based on the kinectomes, we first considered two gait cycle registrations for each individual, called “test” and “retest,” respectively. We then obtained the IM through Pearson's correlation between the test and the retest of our subjects (Figure 1D). The main diagonal of this matrix contains the similarity between two separate acquisitions of the same subject (self‐similarity or I‐self); the off‐diagonal elements contain the similarity between different subjects (I‐others). Furthermore, the difference between I‐self and I‐others, also known as differential identifiability (I‐diff), provides a robust score of the overall fingerprinting assessment of a dataset. Finally, we estimated the identification rate (IR) as the percentage of times a subject was identified when compared to different subjects, as:

$$IR\;=\frac{\sum_{n=1}^{N}\left(Iself_{n}>Iothers_{n}\right)}{N}\;,$$

where *N* is the sample size of the group, Iself_n_ is the similarity between two connectomes of the same individual, and Iothers_n_ is an array of elements representing the similarity between an individual with every other individual of the same group.

The fingerprint analysis assesses the uniqueness of the individual movement patterns. Moreover, if applied within a longitudinal framework, our approach might show individual changes over time.

### Edge‐based identification

We repeated the IR analysis on subsets of edges, based on their contribution to fingerprinting. To obtain this information, similarly to Sorrentino *et al*.,^34^ we used the intraclass correlation (ICC):^45^

$$r\;=\frac{MSA-MSW}{MSA+\left(k-1\right)*MSW}\;,$$

where MSA is the among‐clusters mean square, MSW is the within‐clusters mean square, and *k* represents the number of observations.^45^

It is an approach that assesses how stable an edge value is across test‐retest kinectomes. The higher the stability of an edge between the two kinectomes, the higher the contribution to identifiability. We performed this analysis in PD and HC groups separately, obtaining two ICC matrices. Based on this information, we calculated the IR of the two groups at each step utilizing an iterative model in which we added the edges in descending ICC order from the most to the least contributing to the identifiability. We started with the three edges and kept adding one edge at each iteration, up to including the complete kinectome. We obtained a curve displaying the IR of each group each time an edge was added to the analysis. To confirm the validity of the chosen ordering, for each curve of IR based on ICC ordered edges, we built 100 null curves obtained by calculating the IR based on randomly selected edges. To highlight the nodes that significantly contribute to subject identifiability, we checked how many edges were needed to exceed the 99% IR, and considered those edges as of interest. Then, we checked the distribution of the occurrences of the nodes over which the edges of interest hinge. Nodes whose occurrences exceeded chance level (confidence interval set to 99%) were considered significant and were considered for further investigation. This approach highlights which elements of the motor patterns are specific to the single subject (hence allowing identification). When applied to diseased motor patterns, this analysis may point to anatomical/functional elements of clinical interest.

### Topological feature for the motor impairment prediction

We conceptualized the body as a network, where body parts are nodes and their correlations form the edges, thereby obtaining one weighted undirected graph per subject (Figure 1E). For each node of a graph, we estimated the weighted degree (*s*), a centrality parameter,^13^, ^46^ defined as the sum of the absolute value of the edge weights for each node:^47^

$$s_{i}=\sum_{j=1}^{\begin{array}{c} i\neq j\\ N \end{array}}w_{ij},$$

where *i* and *j* are two nodes of the network, *w* is the edge connecting them, and *N* is the number of nodes.

The degree of the nodes of interest was used to predict clinical impairment in patients. To this aim, we built a multilinear regression model to predict the UPDRS scores from the degree of the nodes of interest.^48^ We added further predictors to the analysis to account for the effect of age, education, and gender. Multicollinearity was assessed through the variance inflation factor.^49^, ^50^ Furthermore, we improved the robustness of our approach using the *k*‐fold cross‐validation, with *k* = 5.^51^ In particular, *k* iterations were performed and at each iteration, the *k*th subgroup was used as a test set. Topological analysis elucidates the role of a single element with respect to the whole body. If a node presents an altered degree (with respect to healthy individuals), then that anatomical element may be of particular interest to understand how the disease affects the motor patterns.

### Statistics

Statistical and data analysis were carried out in MATLAB 2020a. Significance of the between groups (PD and HC) differences in the kinectomes standard deviation, fingerprint values (I‐self, I‐other, and I‐diff), and topological parameter (degree) were assessed through permutation testing, by randomly shuffling group labels 10,000 times. At each permutation, the absolute value of the difference was computed, obtaining a distribution of the differences that are to be expected by chance alone.^52^ This distribution was compared to the observed differences to retrieve a statistical significance. Correlation analysis between nodal degree and motor scores was performed through the Spearman correlation test. The significance threshold was set at *p* < 0.05, and was Bonferroni corrected in each analysis.

## RESULTS

### Group‐specific characteristics of the kinectomes

**FIGURE 3:**
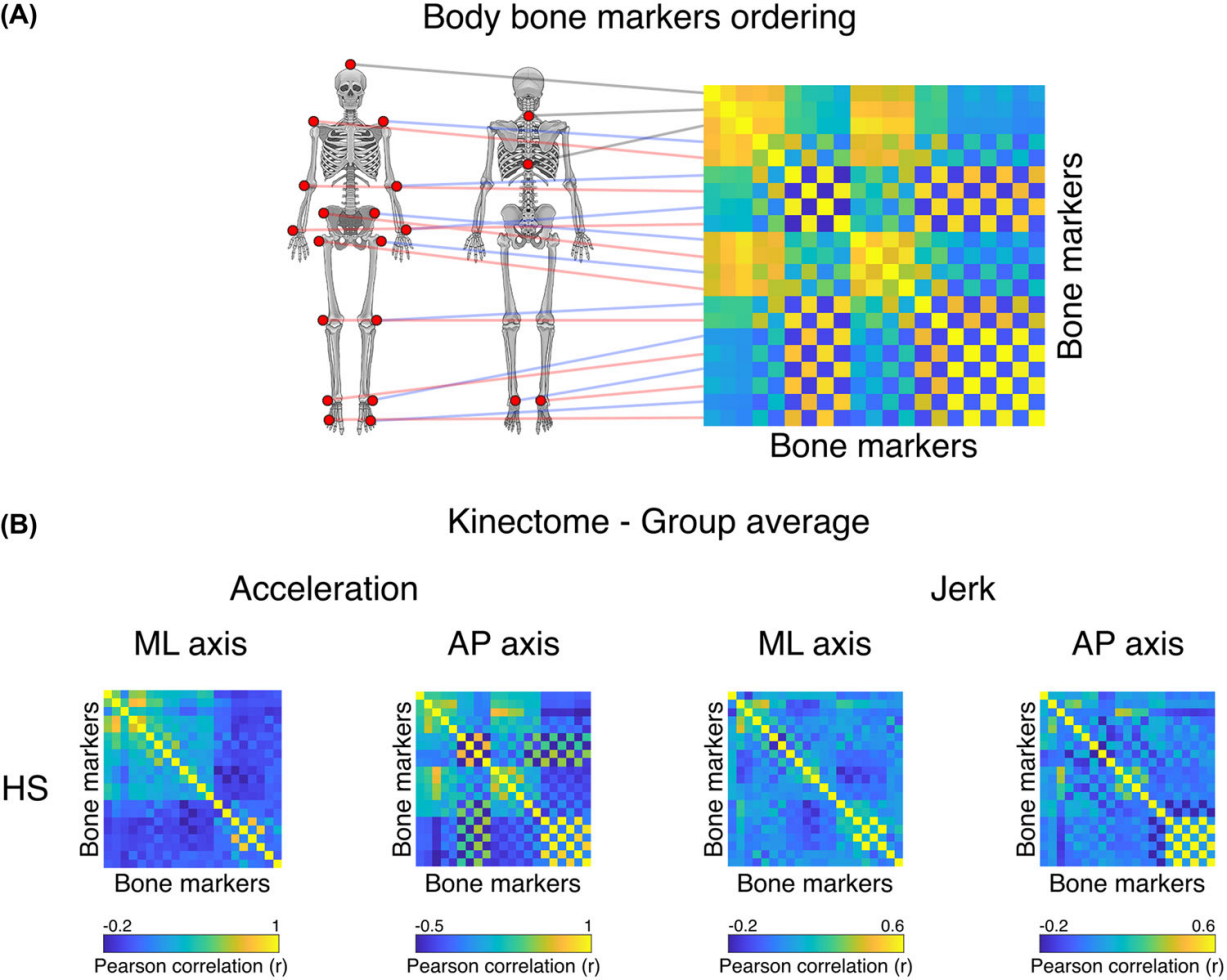
From bones to kinectomes. (A) Illustration of the bone markers position on the kinectome. Each kinematic information derived from each bone marker is used as entry data for both rows and columns. The edges of the kinectome stem from the pairwise interaction between bone markers. (B) Acceleration and jerk kinectomes averaged among healthy subjects (HS) in the mediolateral (ML) and the anteroposterior (AP) axes. The interactions between body elements vary according to both the specific axis and measurement (acceleration or jerk).

We started from a group‐level analysis comparing the average kinectomes of HS and those of PD patients that were recorded during gait. Specifically, after building the subject‐specific kinectomes (Figure 3A), we averaged them within each group, obtaining the group‐specific (i.e., HS, HC, and PD) kinectomes. Then, using permutation analysis, we compared the average values of the kinectomes in HC and PD patients (Figure 3B). However, neither acceleration nor jerk kinectomes highlighted any significant difference between the two groups in any axes of movement. That is, the acceleration and jerk patterns of the two groups were similar to each other. Note that Figure 3B only shows the mediolateral and anteroposterior kinectomes of the HS group. The full set of kinectomes of each group is shown in Supplementary Materials (Figures S1 and S2).

**FIGURE 4:**
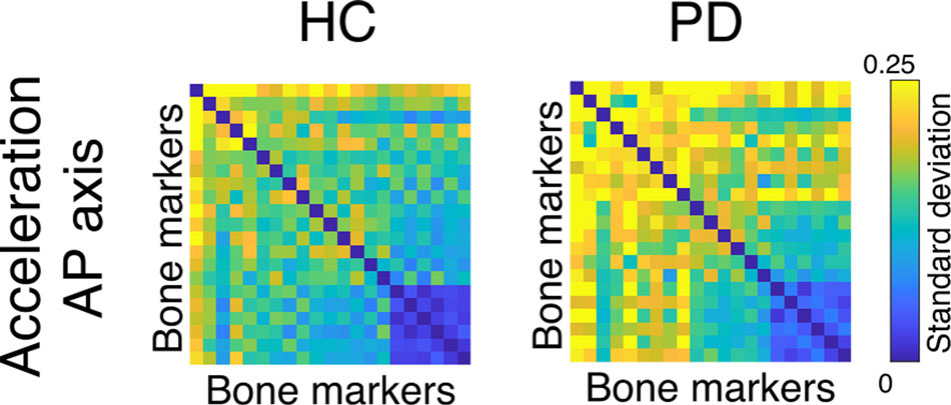
Within group variability of the kinectomes. Standard deviations of the anteroposterior acceleration kinectomes of healthy controls (HC) and Parkinson's patients (PD). Higher values (i.e., yellow entries in the matrices) indicate greater heterogeneity (i.e., higher standard deviation).

Next, we checked the kinectomes’ within‐group variability, by observing the standard deviation of the kinectomes across HS, HC, and PD patients (Figure 4). Notably, the variability in the whole‐body movement patterns between the two groups (HC and PD) showed significantly higher standard deviation among PD patients in the anteroposterior acceleration (*p* = 0.0002, Bonferroni cutoff *p* < 0.0083 over six comparisons, i.e., ML/AP/V jerk and acceleration comparisons), when compared to the HC group (Figure 4). This suggests augmented variability in whole‐body movement patterns for the PD population, which might be due to suboptimal motor control. No significant differences have been found in mediolateral and vertical axes. The full set of standard deviation kinectomes of the three groups (HS, HC, and PD) is shown in Supplementary Materials (Figures S3 and S4).

### Modularity analysis

**FIGURE 5:**
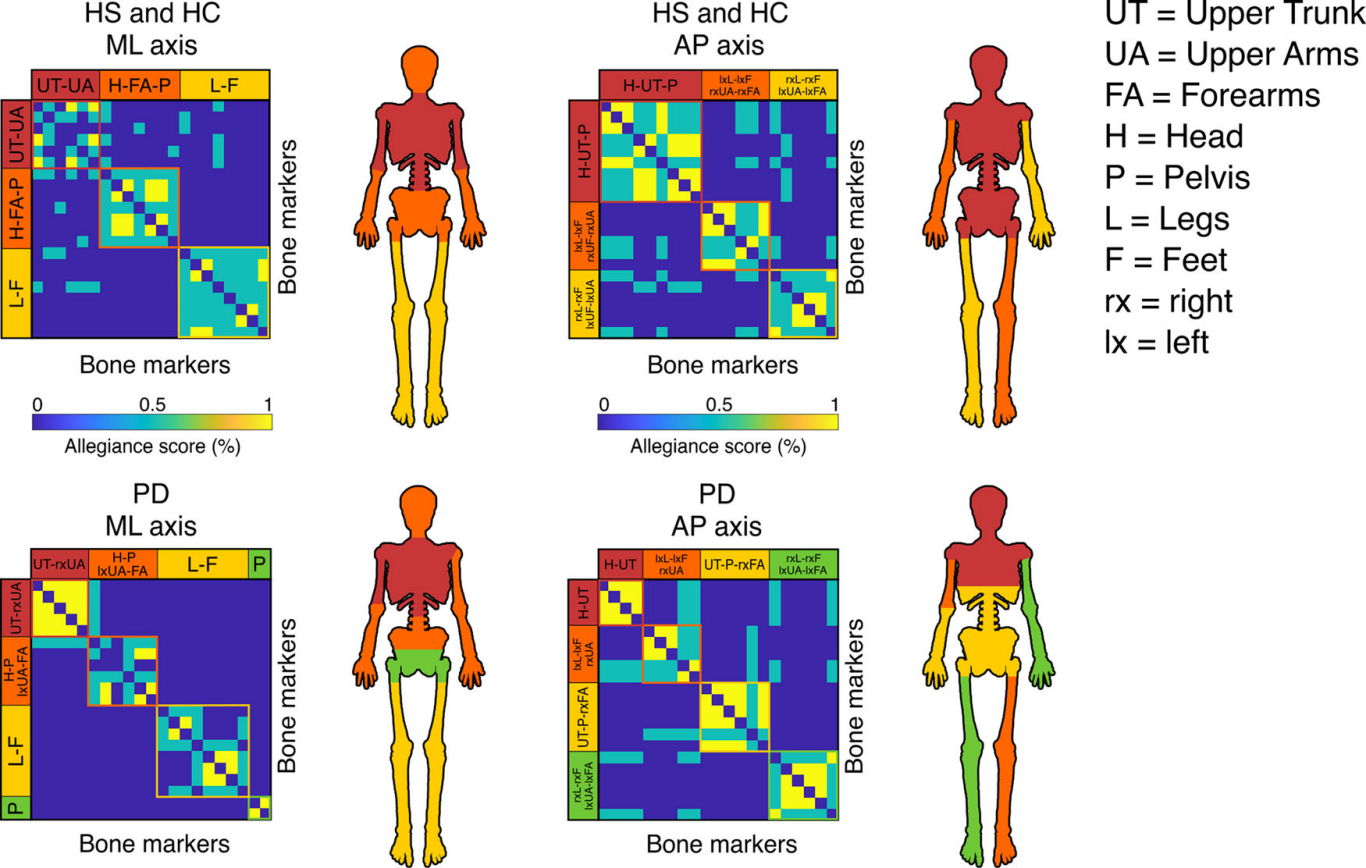
Kinematic modular organization of the kinectomes. Allegiance matrices for cluster analysis, based on the Louvain method and consensus‐clustered through 100 iterations. The algorithm automatically defines which body parts belong to the same community, suggesting a functional relationship among those elements. Each matrix includes clustering information from both accelerations and jerks. Healthy subjects (HS) and healthy controls (HC) share the same communities in both mediolateral (ML) and anteroposterior (AP) axes. Parkinson's disease (PD) patients’ matrices show different structural organizations. Body parts depicted with the same color belong to the same functional community.

We then set out to provide a principled description of the kinectomes’ topological structure, by investigating the emerging modular structure of the kinectomes. To this end, we computed the allegiance matrices,^44^ which contain the probability of any two bone markers being clustered in the same community across individuals. This means that two or more bone markers belonging to the same cluster refer to body parts which are likely to coordinate themselves toward the same motor pattern, for each specific group. Figure 5 shows that the HS and HC groups share the same communities, while the PD group features a different clustering pattern. Specifically, in the healthy groups, the ML allegiance matrix showed three communities: (1) upper trunk and arms; (2) head, forearms, and pelvis; and (3) legs and feet. In the PD group, the same matrix showed four different communities: (1) upper trunk and right upper arm; (2) head, left upper arm, forearms, and pelvis (upper portion); (3) legs and feet; and (4) pelvis (lower portion). The AP allegiance matrix in the healthy groups highlighted three communities: (1) head, upper trunk, and pelvis; (2) left leg and foot, and right upper arm, and forearm; and (3) right leg and foot, and left upper arm and forearm. In the PD group, the same matrix identified four communities: (1) head and upper trunk (upper portion); (2) left leg and foot, and right upper arm; (3) upper trunk (lower portion), pelvis and right forearm; and (4) right leg and left upper arm and forearm. Finally, the modularity analysis concerning the vertical axis is shown in Supplementary Materials (Figure S5). In this case, the healthy groups presented the following communities: (1) head, trunk, pelvis, and legs; (2) arms; and (3) feet. The PD patients presented the same clusters with a slight difference that sees the left side of the pelvis being included in the “arms” community.

This approach unraveled the kinematic structure of gait in the healthy, and its alterations in disease. It is noteworthy that the algorithm calculating the communities split the body parts symmetrically in healthy individuals, while the same result was not achieved for the PD patients, which might be due to the typically asymmetrical motor impairments occurring in PD, which is even a diagnostic criterion.

### Fingerprint of human movement

**FIGURE 6:**
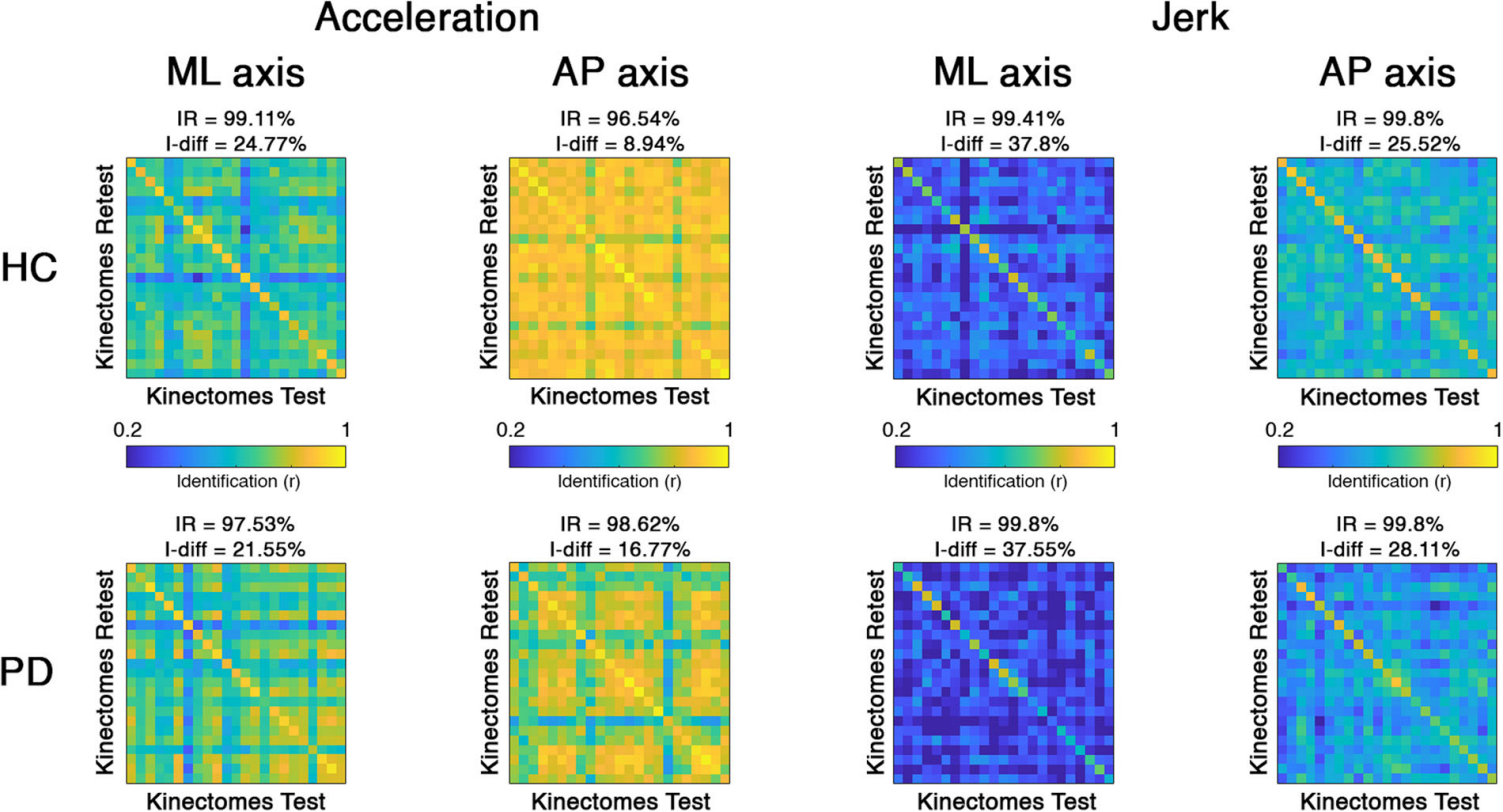
Motion fingerprinting: identifiability based on kinectomes. Identifiability matrices of healthy controls (HC) and Parkinson's disease (PD) patients, based on jerk and acceleration kinectomes in mediolateral (ML) and anteroposterior (AP) axes. The highest values within the main diagonal (I‐self) convey great self‐similarity. Off diagonal elements (I‐others) are representative of the similarity between different subjects. IR, identification rate; I‐diff is the differential identifiability scores of the dataset and is defined as I‐self–I‐others.

Based on these results, we wondered whether it was possible to identify each individual based on their motion patterns. To answer this question, we tried to identify individuals through their kinectomes, obtained from different gait sessions recorded the same day. To this aim, we started by building an IM based on the kinectomes.^33^ In the IM, the rows refer to the kinectomes from the first recording session (test kinectomes in Figure 1D), and the entries on the columns refer to the kinectomes from the second recording session (retest kinectomes in Figure 1D). The entries of the IM are Pearson's correlation coefficients between the kinectomes derived from the first and the second recording sessions. Briefly, from the IM, we calculated three parameters: the I‐self (self‐similarity, across the two recordings of the same individual), the I‐others (similarity with other individuals within the group), and the I‐diff (differential identifiability), obtained subtracting the I‐other from the I‐self. The I‐diff expresses how much an individual is recognizable with respect to the other individuals. Figure 6 displays the acceleration and jerk IM for both ML and AP axes, in PD and HC groups, while information on the HS group and the vertical axis is shown in Supplementary Materials (Figures S6 and S7). AP and ML jerk were the best quantities for gait identifiability (highest I‐diff values), and this result applies to both groups (I‐diff > 37.5%). Beyond the I‐diff, the identifiability rate (IR – the percentage of times the I‐self of an individual is higher than any I‐others, i.e., the two kinectomes belonging to the same individual are most similar to each other as compared to the kinectomes belonging to any other participant) is higher than 95% for each parameter in each direction of movement. Strikingly, this approach allowed subject recognition from gait relying on approximately 2‐s long recordings. Hence, our approach nearly always correctly identifies the individuals, regardless of them being HS or patients. For further validation, the IM was computed in the HS group, where it confirmed the optimal performances showed in the HC group (Figure S6). Hence, expanding the sample (from 23 to 60 subjects) did not affect the performance of the fingerprinting approach.

**FIGURE 7:**
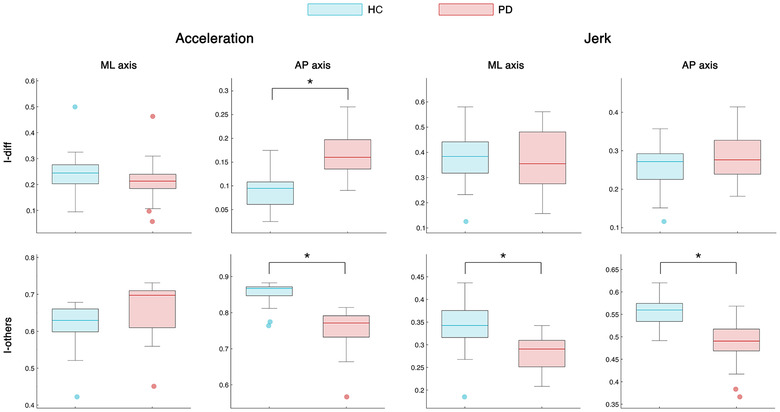
Identifiability comparison between healthy controls and patients. Box plot for the comparison of I‐diff and I‐others between healthy controls (HC) and patients with Parkinson's disease (PD). High I‐diff values imply that individuals are more similar to themselves than they are to the other subjects of the same group. High I‐others values indicate high within‐group similarity among the subjects of a group. The box represents data from the 25th to the 75th percentiles; the horizontal line shows the median; error lines indicate the 10th and 90th percentiles, and values falling beyond them are represented by colored dots. *, represents significant Bonferroni‐corrected *p*‐values.

Comparing PD and HC groups (permutation test, Bonferroni cutoff *p* < 0.0028 over 18 comparisons, i.e., I‐self, I‐others, and I‐diff in AP/ML/V jerk/acceleration kinectomes), no difference was found in the I‐self parameter, nor in any analysis comparison involving the vertical axis. However, with respect to the I‐diff scores, the PD group showed higher values compared to the HC group in AP acceleration axis (*p* < 0.0001) (Figure 7). This effect is mainly driven by the difference in the I‐others scores. In fact, the PD group showed lower I‐others scores as compared to HC patients in AP acceleration (*p* < 0.0001), ML jerk (*p* < 0.0001), and AP jerk (*p* < 0.0001) (Figure 7). This implies that the PD patients have more heterogeneous motor patterns with respect to the HC groups; hence, their kinectomes differ more with respect to each other. Nonetheless, both groups expressed similar IRs, which were above 95%. This result highlighted that an almost perfect identification is possible for both PD patients and controls.

### Edges contribution to subjects’ identification

We wondered if the loss of coordination observed in PD is a generalized phenomenon or, rather, affects the interactions of specific body segments. In the latter case, the different IR between the PD and HC groups should be due to a subset of specific edges. To test this, we calculated the IR iteratively, using each time a different number of edges (from 3 to 210) ordered from the highest to the lowest ICC rank. Based on this, we built an IR curve describing the *identification rate* as a function of the number of added edges, as described. The analysis was performed in each group (PD and HC) separately, and for each parameter (AP/ML/V acceleration and AP/ML/V jerk). To confirm the reliability of our approach, each step of the curve was validated using a null model based on 100 IRs, each calculated on the same number of randomly chosen edges (Figure S3). For each IR curve, in both HC and PD, the null‐models performed worse than the ICC‐ordered IR curve. This result talks to the clinical validity of the subset of selected edges since the same quantity of randomly selected edges does not perform as well. In other words, identification is based on a subset of edges, which are the ones representing the most fine‐tuned interactions. In fact, in both groups, the whole kinectome is not needed to maximize identifiability. Rather, a small subset of edges is enough, and this set is smaller in HC with respect to PD patients. In fact, we observed that, in order to exceed the 99% identifiability threshold, the HC group needed approximately 12 edges on average, while the PD group needed approximately 30 edges. Again, this is in line with a dysregulation of the interactions in PD.

### Nodal relevance for clinical evaluation

Following the previous analysis, we focused on the 30 edges with highest ICC value of the PD group. Indeed, since those edges were sufficient to maximize the identifiability of each patient for each parameter (AP/ML acceleration and AP/ML jerk), we assumed that they carried most subject‐specific information. Hence, we counted, for each parameter, how often (across subjects) the 30 edges would be incident on any given node (i.e., body element). We observed that the T10 node in the ML acceleration (MLA‐T10, the 10th thoracic vertebra) was included above chance level by the 30 edges (falling outside the upper limit of the 1–99% confidence interval). Subsequently, for the ML acceleration kinectome of each subject, we calculated the weighted degree (the sum of the weights of all the edges incident upon a given node) of the T10 node (Figure 9A, left panel). Comparing the MLA‐T10 degree between HC (5.07 ± 0.96) and PD (6.22 ± 1.7), we observed that the patients showed significantly higher values (*p* = 0.0069) (Figure 9A, middle panel). That is, the PD group showed higher degree at thoracic level during mediolateral acceleration movements. To test the clinical relevance of this finding, we used the Spearman correlation to investigate associations between the degree of MLA‐T10 and the clinical condition of PD patients, evaluated through UPDRS. We found a significant positive correlation (*r* = 0.65, *p* = 0.0007), meaning that the higher the motor impairment, the higher the degree of MLA‐T10 (Figure 9A, right panel). This might be capturing the rigidity, a typical clinical feature in PD.

### Network‐based clinical prediction

We then wondered whether MLA‐T10 could predict patient‐specific motor impairment. To this end, we performed a multilinear regression analysis to predict the UPDRS scores based on the MLA‐T10 degree values.^48^ We included three nuisance variables in the regression model to account for confounds, such as age, sex, and education. The prediction model was validated through *k*‐fold cross validation (*k* = 5),^51^ to test its specificity and generalization capacity. We found that the model based on the MLA‐T10 degree significantly predicts the UPDRS (*p* = 0.003, *R* ^2^ = 0.44) with a positive beta coefficient (Figure 9B, left panel). That is, the higher the degree, the higher the UPDRS score. None of the remaining predictors was significant. The agreement of the actual/predicted UPDRS scores, and the distribution of the residuals can be observed in Figure 9B (middle and right panels, respectively).

## DISCUSSION

**FIGURE 8:**
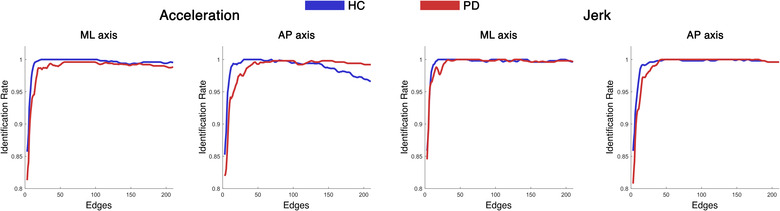
Edge‐based identification rate. Identification rate (IR) for healthy controls (HC) and patients with Parkinson's disease (PD) kinectomes, for acceleration and jerk parameters in mediolateral (ML) and anteroposterior (AP) axes. The IR is computed in an iterative fashion: starting from three edges, at each iteration, one edge is added and the IR is computed. The edges were included following an order based on their contribution to the identifiability (from the most to the least contributing), as measured by the intraclass correlation analysis. The HC group exceeded the 99% identification threshold with a smaller number of edges (roughly 12), as compared to the PD patients (about 30).

**FIGURE 9:**
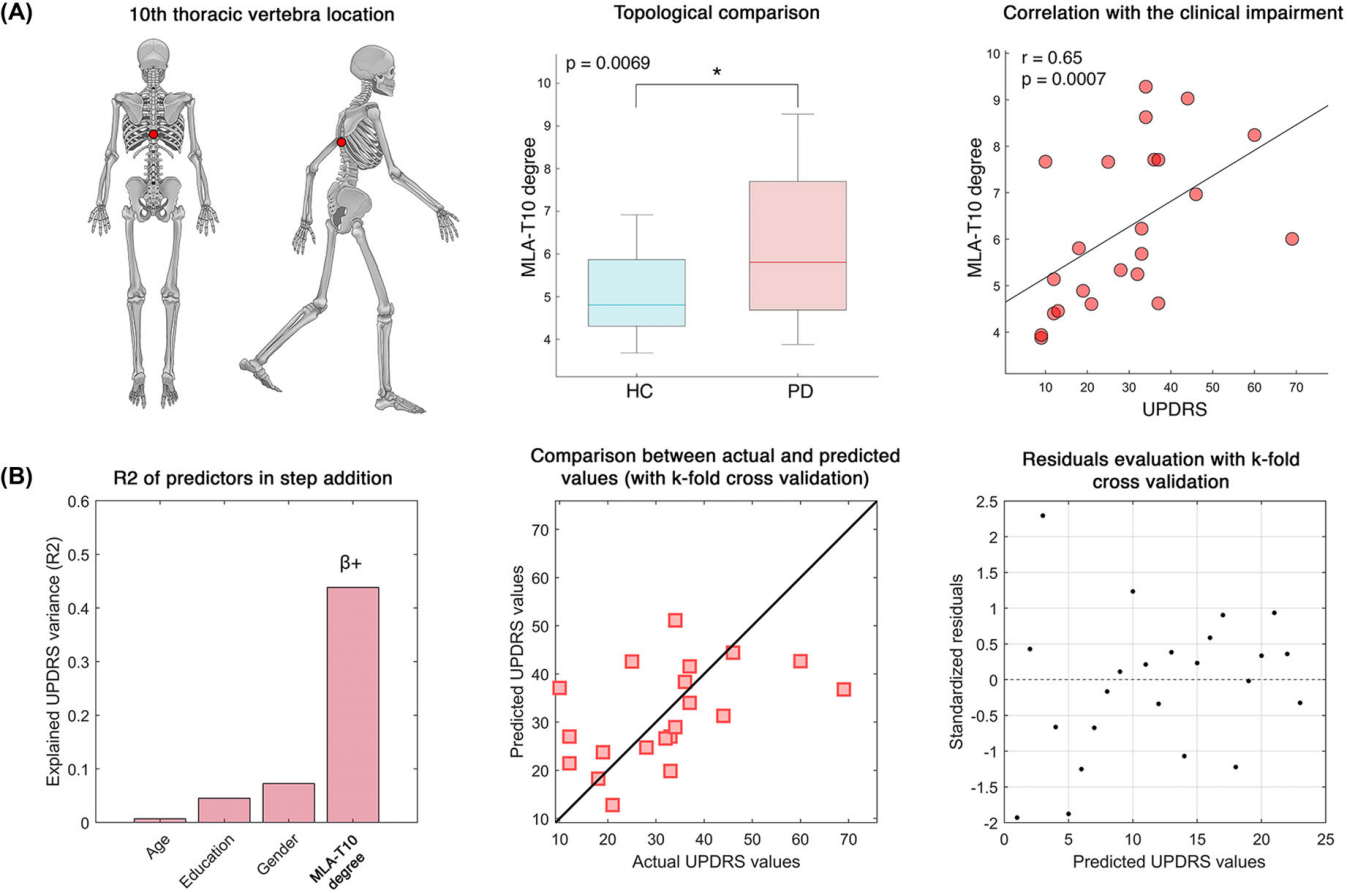
Clinical relevance of kinectome features. (A) The left panel highlights the position of the 10th thoracic vertebra, whose degree in the kinectome derived from the mediolateral accelerations (MLA‐T10) has been analyzed within a clinical framework. The middle panel shows that patients with Parkinson's disease (PD) have significantly higher MLA‐T10 degree with respect to the healthy controls (HC); the right panel shows the positive significant correlation between the MLA‐T10 degree and the clinical motor impairment assessed through the Unified Parkinson's Disease Rating Scale (UPDRS). (B) Multilinear regression model for the prediction of the UPDRS from the MLA‐T10 degree. The left panel shows the explained variance (*R* ^2^) of the UPDRS, while sequentially adding the predictors (i.e., age, education, gender, and MLA‐T10 degree) to the model; MLA‐T10 degree was a significant predictor with positive beta coefficient; the middle panel displays the relationship between empirical and predicted UPDRS scores, with *k*‐fold cross validation (*k* = 5); and the right panel illustrates the distribution of the residuals with *k*‐fold cross validation (*k* = 5).

In this paper, we propose a novel approach to analyze human movement, based on the kinectome, a mathematical structure containing the pairwise interactions between different body segments during gait. In fact, the kinectome consists of the covariance matrix of the accelerations (or the jerks) of all body segments. First, we show that the kinectome provides a thorough description of gait and distinguishes population‐specific features (Figures 3 and 4). Second, the kinectome captures symmetries in the modularity of the human motion patterns, which are lost in PD patients (Figure 5). Third, through the kinectome analysis, it is possible to identify subjects based on their gait data, using only short (∼2 s) recordings (Figures 6, 7, 8). Finally, and most importantly, the topological analysis of the kinectomes allows us to explore the role of individual biomechanical elements of the human kinematic network within a holistic, complex system perspective, and to use this information to predict clinical impairment (Figure 9). These findings confirm the potential of the kinectome in tapping into the complex dynamics arising during human movement and their alteration in disease.

The presence of groups of functionally related body parts emerged naturally from the analysis of the kinectome, showing that the covariance of accelerations conveys biomechanically meaningful information. Furthermore, these patterns have clinical relevance, since they differentiate healthy individuals from patients with PD. Notably, the best distinction between the groups was obtained using the variance of the acceleration (Figure 4), with patients having much more variability as compared to the controls, and particularly so in the upper body along the anteroposterior axis. We speculate that the healthy patterns of movements are optimally constrained, especially in the upper body segments (which are very relevant to allow bipedal locomotion).^5^ At the same time, the neurological impairment in PD compromises the motor control, which manifests itself in more variable, dysregulated gait patterns.^53^ These results are in line with the literature.^32^ In fact, several studies confirmed greater variability of gait in PD patients as compared to controls, using a number of metrics and techniques. A recent review considered several studies of gait analysis in PD patients, with the aim of describing gait impairment, the underlying mechanisms, and the relationship to disease progression.^31^ From the data collected by Mirelman *et al*., it is evident that increased variability is one of the most common findings in PD patients. However, the authors also pointed out the need for standardized practice for variability assessment. Our approach, which is applied at the subject‐level, may highlight the variability across the whole body over different trials.

From the biomechanical perspective, the clustering analysis of the kinectome reveals the large‐scale functional organization of the body segments (Figure 5). It is our opinion that the ML allegiance matrix highlights the stabilization mechanism occurring during mediolateral oscillations in HS. During gait, the swing phase reduces the width of the base of support, decreasing the stability. Pelvis smoothens the movement of the center of mass,^54^, ^55^ as well as forearms counterbalance its displacement contributing to the vertical alignment between head and pelvis.^56^ Finally, the upper trunk produces controlled oscillations in order to stabilize the head.^57^, ^58^ However, the modularity patterns are consistently rearranged in PD patients. With regard to the PD allegiance ML matrix, the modules that were observed in the healthy individuals are altered in PD, where body movements appear more fragmented. Indeed, unlike the healthy modules, the left and right upper arms belong to two different modules in PD, and the same goes for the pelvis (upper and lower pelvis do not belong to the same functional module). These alterations may be due to postural abnormalities and asymmetries, features that are typically altered in the gait of PD patients.^30^, ^59^ In particular, asymmetry is widely studied in PD, as it is one of the clinical hallmarks of the disease. Several approaches have been used to quantify asymmetry, most of which focused on the right‐to‐left ratio. Recently, Godi *et al*. quantified the power of asymmetry in discriminating HS from individuals with PD, based on the absolute value of the natural logarithm of the left‐to‐right ratio.^60^ Another approach, performed by Orcioli‐Silva *et al*., evaluated the laterality as the ratio between the sum and the difference of the dominant and the nondominant side.^61^ In their study, the authors suggested that, in the presence of obstacles, the asymmetry in PD becomes more pronounced. Despite highlighting the asymmetry (among other elements), our approach is different in nature, as it groups body elements in a data‐driven fashion, based on their acceleration patterns.

With regard to the AP axis, the allegiance matrix of the healthy groups distinguished the passenger unit from the locomotor unit.^62^ The former is composed of the head, the trunk (including the pelvis), and the arms, the latter includes the lower limbs. However, our analysis grouped together the accelerations of the arms and the legs, defining two separate communities encompassing contralateral arms and legs. This shows the fact that our approach goes beyond purely anatomical arguments, and defines modules on functional grounds. In fact, our definition of the modules captures the fact that arms oscillate in antiphase with respect to the contralateral legs.^63^ Interestingly, this linear pattern fails in PD, especially with respect to the trunk. In fact, the first community is composed of the head and the upper trunk in PD, while the lower trunk and the pelvis belong to a different community together with the right forearm. The two remaining communities capture the antiphased oscillations between contralateral arms and legs, as observed in the healthy groups. Once again, we can relate these disrupted patterns to the motor characteristics of parkinsonian patients. On the one hand, the asymmetry may have caused the dysregulation of the acceleration of the right arm with respect to the healthy pattern.^59^ On the other hand, the axial rigidity, a semiologic feature of the disease,^64^ does not allow the trunk to effectively relay multiple body parts. Hence, different subsections of the trunk remain entrained to more peripheral anatomical parts. In turn, this is captured by the fact that the trunk is split in different communities in patients, instead of being a coherent functional unit as seen in the HC group. Once again, the kinectomes allow us to identify the features of gait that are shared by all HS and that are lost in PD patients.

Finally, the modularity expressed in the vertical axis was very similar between HS and PD patients. Verticality in gait is characterized by the raising of the whole body at each step, identified by the clustering involving the whole body, with the exclusion of arms and feet, that in turn were included in two separate communities. The arms community stems from the vertical component of the arm oscillation that goes with walking, while the community encompassing the feet includes those anatomical elements that alternatively engage in the swing phase. In this case, the only difference within the PD clustering was the presence on the left side of the pelvis in the arms community. This result may be attributed once again to the asymmetry of the patients.

The kinectomes can be exploited further, to identify subject‐specific gait features, thus defining a “fingerprint” of the human gait. Our analysis demonstrated that the kinectomes carried a unique pattern for each individual. In fact, using (approximately) 2‐s long acquisitions as test and retest sessions, we were able to identify subjects with an accuracy rate above 99%. Note that the jerk kinectomes were the most reliable in identifying individuals (Figure 6). PD patients exhibited identifiability rates similar to those of the controls. However, the similarity within the PD group (as measured by the I‐others) was lower than that within the control group (Figure 7). In other words, controls are more similar to each other than PD patients. One might speculate that a correct motor control imposes stricter constraints to the kinectome structure, which in turn produces more similar motion patterns. In pathological conditions, such as PD, such control mechanisms would fail, the constraints on the gait pattern would become looser and, hence, the patterns would be less similar to each other. This alteration in motor patterns could be related to the increase in gait variability, which, as mentioned above, is one of the gait features most commonly found in Parkinson's patients.

Next, we started exploring the contribution of individual edges to the identification (Figure 8 and Figure S8). First, it can be noticed that only a few edges suffice for an optimal recognition. Indeed, human movement interactions are fine‐tuned, and only a few are sufficient to define a fingerprint of movement and, hence, to identify individuals (even when they are affected by a motor disease). Beyond this general viewpoint, the edges that mainly contribute to identification in the clinical framework may represent the interactions between the body elements that are mainly affected by the disease (in terms of coordination and control). A possible explanation of this finding stems from the consideration that the way and the extent to which motor behavior is affected is patient specific. This, in turn, might enhance the identifiability of the patterns, making patients more identifiable. The kinectome is effective in capturing these patterns and, accordingly, it shows enhanced identifiability in PD patients. Comparing both HC and patients, the jerk IR models maximized identifiability. Indeed, the IR of patients on the mediolateral and vertical acceleration does not perform as well (compared to the healthy individuals) as in the jerk IR analysis. This outcome may reflect the impaired ability of patients to control mediolateral and vertical movement, thereby generating more variable patterns (at the individual level), which are harder to recognize. With regard to the anteroposterior acceleration, despite a better performance of the healthy individuals when focusing on a few edges, the IR of the controls drops when including all the edges into the analysis. The interpretation of this finding is challenging. We hypothesize that, when all the elements of the body are considered, the healthy individuals manage anteroposterior accelerations similarly (and, presumably, optimally), generating similar patterns that make identification harder. Furthermore, with respect to the HC, the patients needed the contribution of more edges to exceed the 99% identifiability threshold. We hypothesize that healthy individuals are more likely to repeat a motor behavior with high precision, determining well‐defined patterns (based on a few specific edges, highlighted by the ICC analysis) that help recognition. On the contrary, the impaired motor behavior of PD patients may result in dysregulated movement patterns (which is in line with the modularity analysis) that are harder to recognize. Consequently, the contribution of a higher number of interactions is needed for identification.

To reach a clinical interpretation of this finding, we investigated which nodes (i.e., body parts) were involved the most in subject identification in the PD group. We reasoned that, if all that edges upon which subject identification is based preferentially hinge on some node(s), then this node would point at a specifically relevant region for large‐scale coordination, and might optimally capture pathological processes as well. Indeed, the MLA‐T10 node (which occurred the most in edges that allow subject identification, and is representative of the mediolateral acceleration of the 10th thoracic vertebra) showed several interesting features. First, we observed that the MLA‐T10 degree was higher within the PD group as compared to the HC group. As said, this might be capturing the trunk rigidity typical of PD,^65^, ^66^, ^67^, ^68^, ^69^ causing hypersynchronization of the movement between the trunk and the limbs. Several studies focused on trunk kinematics, in particular for the assessment of the smoothness of gait, using different metrics.^32^, ^70^ Lowry *et al*. assessed the walking stability in PD using the harmonic ratio, a measure that takes into account the rhythm of trunk acceleration.^71^ The authors analyzed gait fluidity in PD patients, and showed reduced harmonic ratio over the three axes. Similar results were found by Cole *et al*.^72^ A different approach was presented by Beck *et al*., which used the spectral arc length measure to assess the smoothness of trunk acceleration in PD.^29^ The authors reported reduced smoothness in patients as compared to HC and, notably, a correlation with the UPDRS. All these studies, regardless of the methodological approach, highlighted the trunk acceleration as an informative element, and our results are in line with these findings. Further longitudinal studies may explore the potential of this approach in diagnostics and assessment/prediction of therapeutic responsiveness. Furthermore, we found that the MLA‐T10 degree of the patients was significantly correlated with the motor impairment evaluated through the UPDRS scores (Figure 9A). This correlation showed that the greater the motor impairment, the more the mediolateral accelerations of the upper trunk were coherent with those of the other body segments. Axial rigidity and postural abnormalities are typical features of PD that might reflect themselves into “hyperconnected” patterns.^73^, ^74^, ^75^ Finally, we observed that the MLA‐T10 degree could predict the UPDRS score at individual level, even after taking into consideration confounding variables, such as age, gender, and education. Notably, the prediction has been validated with *k*‐fold cross validation. This result highlighted that the hyper connectedness shown by the mediolateral trunk acceleration was strongly related to the subject‐specific impairment level. This outcome may open the possibility to use this approach to monitor at individual level both the disease progression and the effects of pharmacological therapies and rehabilitation protocols.

It should be stressed that this is the first time that the kinectome is defined. Hence, its ability to convey the individual clinical condition needs to be tested in samples, including more PD patients, as well as both in different movement disorders and in more neurological diseases. In our analysis, it was observed a lack of significant results concerning the vertical axis. One explanation could be that the vertical axis may be less informative when assessing the synchronization of movement during gait. Furthermore, the amplitude of the movements along the vertical axis is smaller as compared to the other direction, thereby making measurements less precise and, hence, less reliable. Methodologically, further analysis should be performed to explore the required number of bone markers for an optimal spatial resolution of the kinectome. In this work, we utilized 21 markers to sample the whole body. However, according to the research question at hand, it may be useful to vary the number and locations of the markers. As an example, one may also consider to focus on specific subnetworks (e.g., the lower limb network), as appropriate to test specific working hypotheses. Finally, in this work, we only consider pairwise interactions, future work should also consider higher‐order interactions.^76^

In conclusion, we have proposed a network approach to motion analysis, which identified several disease‐related features, both at global and individual levels. It must be noted that the methodology underlying our approach is grounded in network science, which is a solid branch of mathematics. We believe that the application of such methodology in the motion analysis opens new possibilities, especially in the domain of clinical applications, where it allows disease‐specific motor features examination. In particular, we think that the kinectome represents an informative tool, which encodes a large number of features that can be extracted, depending on the specific research question at hand. In this work, we started from a global examination, we then zoomed in to describe the characteristics of the kinectome that emphasize individual features, up to revealing a kinematic element (i.e., the MLA‐T10), which captures many of the significant features of PD. Further studies may focus on the usefulness of exploiting the kinectome to support pharmacological and rehabilitative treatments. Finally, this study limited itself to a first application, but the kinectome is a powerful tool that can be applied in a wide spectrum of frameworks. Useful applications could be in the differential diagnosis between PD and atypical parkinsonisms (e.g., multiple system atrophy, progressive supranuclear palsy, and corticobasal syndrome). More in general, this approach might be useful to capture pathophysiological changes that manifest in subtle alterations of the interactions among multiple body parts. These kinds of patterns are easily missed by the human eye, and might greatly help doctors to refine diagnosis by taking this information into account. Finally, other fields of applications will be in biomedical engineering for the construction of robots, or to design motor assistance devices.

## AUTHOR CONTRIBUTIONS

Conceptualization: E.T.L., P.S., E.A., and G.S.; methodology: E.T.L., E.A., G.S., M.L., and R.M.; software: E.T.L., P.S., and E.A.; formal analysis: E.T.L., P.S., and E.A.; investigation: E.T.L., M.L., R.M., A.R., and A.P.; resources: A.C. and G.S.; data curation: E.T.L., M.L., R.M., A.C., A.R., and A.P.; writing—original draft: E.T.L., P.S., E.A., and G.S.; supervision: E.A. and G.S.; project administration: G.S.; funding acquisition: E.A. and G.S.; and writing—review and editing: all authors.

## COMPETING INTERESTS

The authors declare no competing interests.

### PEER REVIEW

The peer review history for this article is available at https://publons.com/publon/10.1111/nyas.14860.

## Supporting information

Additional supporting information may be found in the online version of the article at the publisher's website.
