# Supplementary material for: The kinectome: A comprehensive kinematic map of human motion in health and disease
Source: Ann N Y Acad Sci. 2022 Jul 15;1516(1):247–61. doi: 10.1111/nyas.14860 (PMC9796708; doi:10.1111/nyas.14860)
Supplement: Supplementary file 1 — Additional supporting information may be found in the online version of the article at the publisher's website. [file NYAS-1516-247-s001.docx]

**Supplementary materials**

**
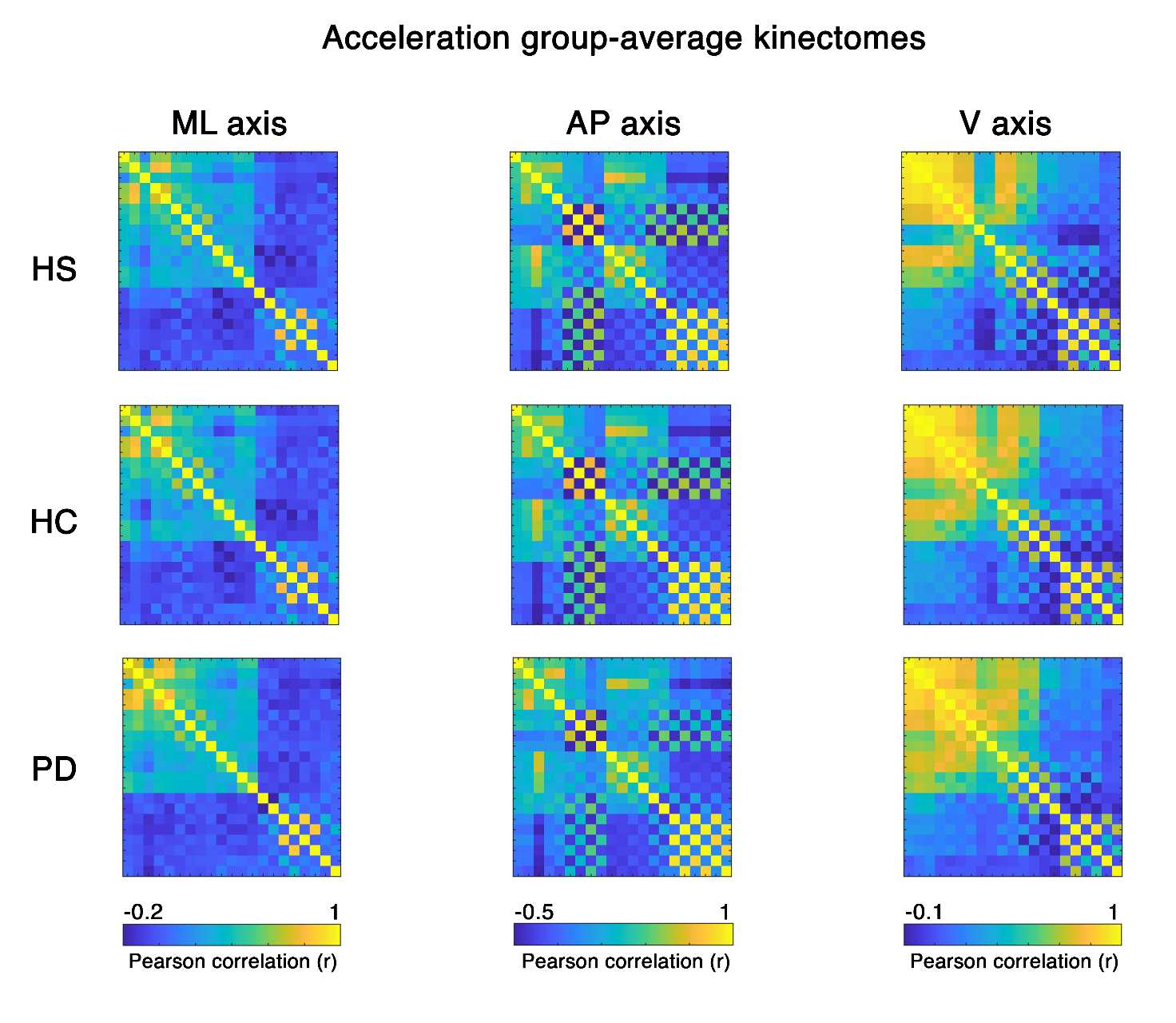
**

**Figure S1. Group average acceleration kinectomes**

Averaged acceleration kinectomes for healthy subjects (HS), healthy control group (HC) and Parkinson’s disease group (PD). Kinectomes are shown for mediolateral (ML), anteroposterior (AP), and vertical (V) axes. Despite slight differences, the kinectomes are similar among the three groups, and no statistically significant difference was found.

**
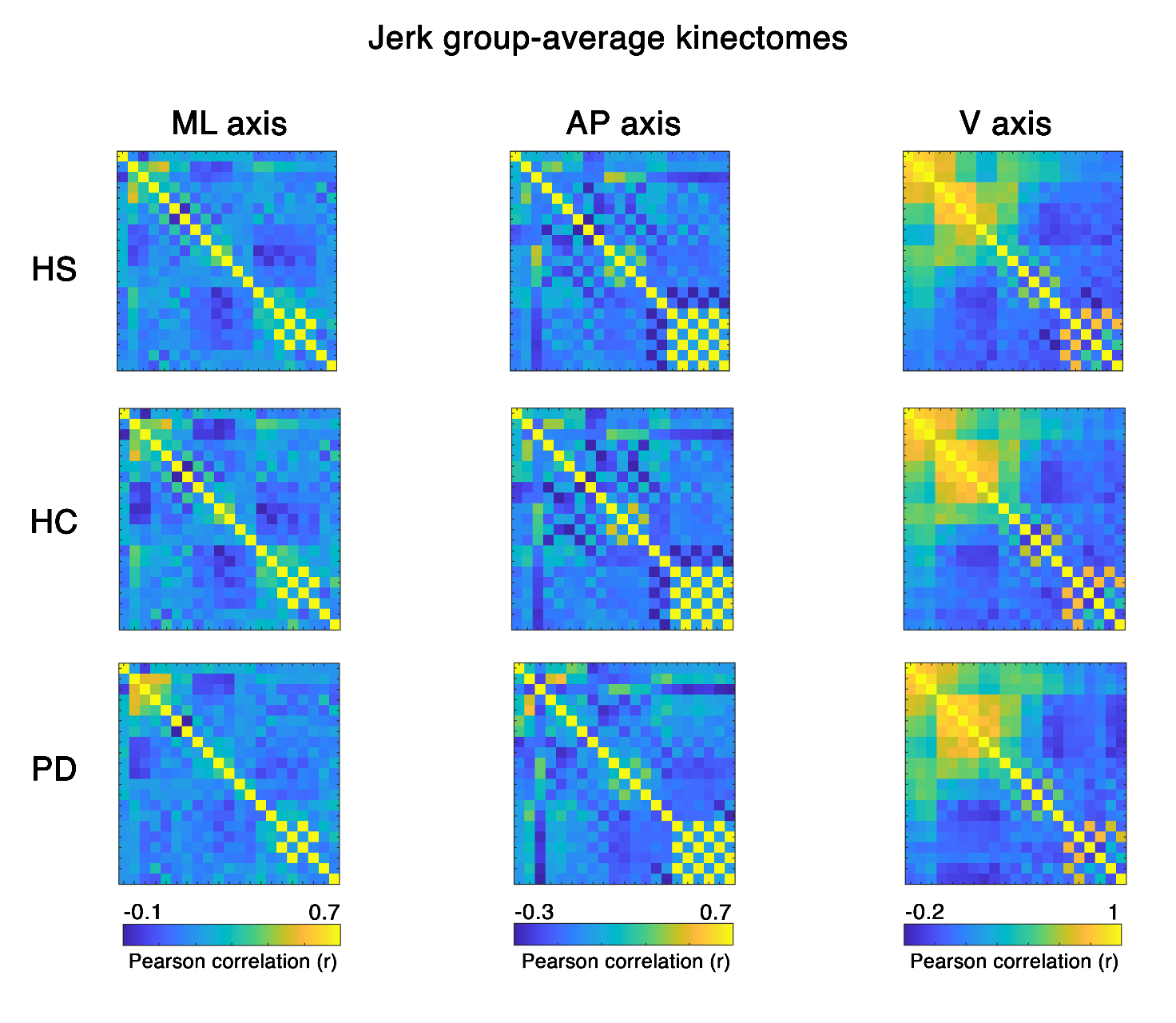
**

**Figure S2. Group average jerk kinectomes**

Averaged jerk kinectomes for healthy subjects (HS), healthy control group (HC) and Parkinson’s disease group (PD). Kinectomes are shown for mediolateral (ML), anteroposterior (AP), and vertical (V) axes. Despite slight differences, the kinectomes are similar among the three groups, and no statistically significant difference was found.

**
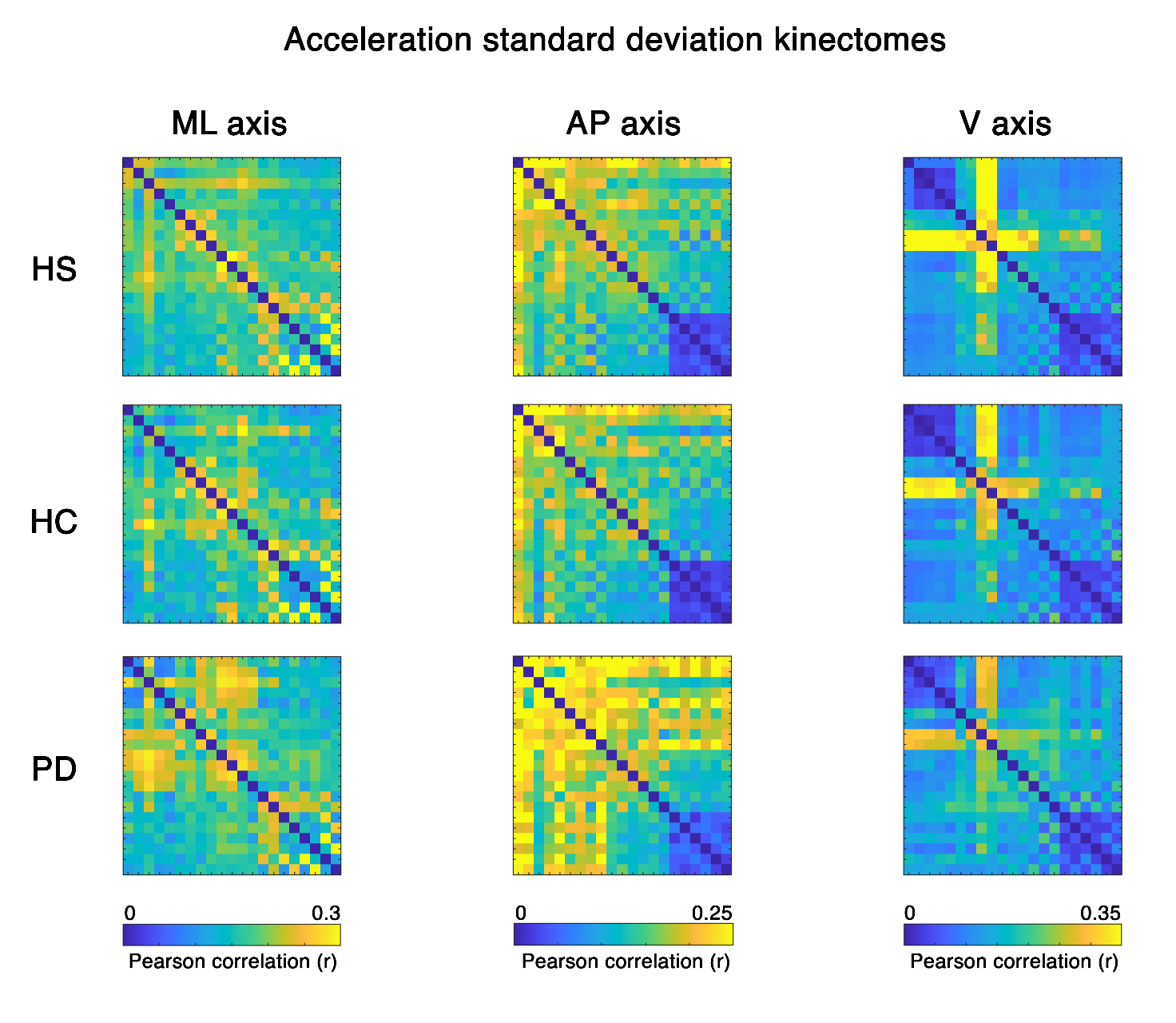
**

**Figure S3. Within-group standard deviation of the acceleration kinectomes**

The figure represents the within-group variability among acceleration kinectomes. It includes information on healthy subjects (HS), healthy control group (HC) and Parkinson’s disease group (PD), in mediolateral (ML), anteroposterior (AP), and vertical (V) axes.

**
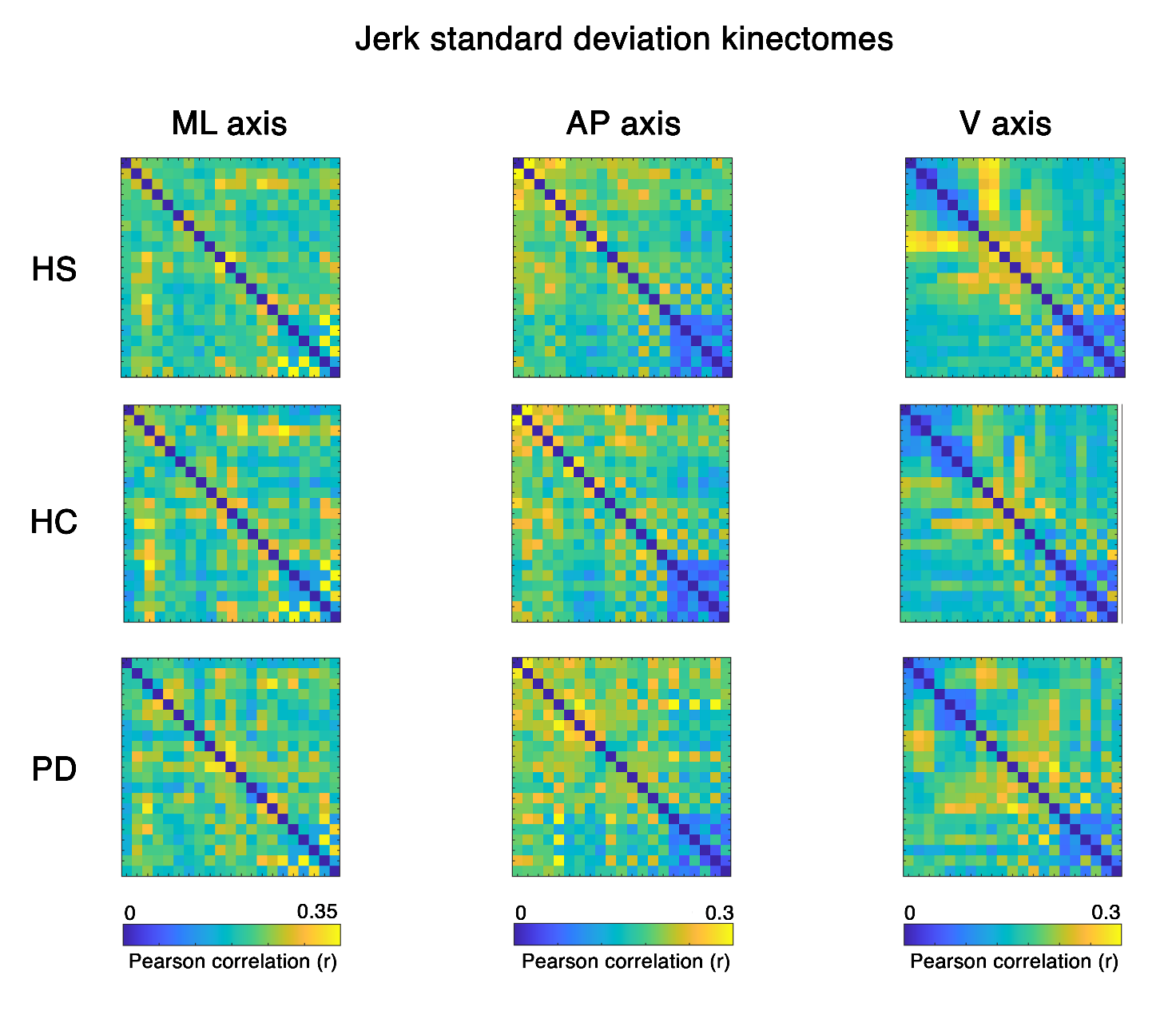
**

**Figure S4. Within-group standard deviation of the jerk kinectomes**

The figure represents the within-group variability among jerk kinectomes. It includes information on healthy subjects (HS), healthy control group (HC) and Parkinson’s disease group (PD), in mediolateral (ML), anteroposterior (AP), and vertical (V) axes.


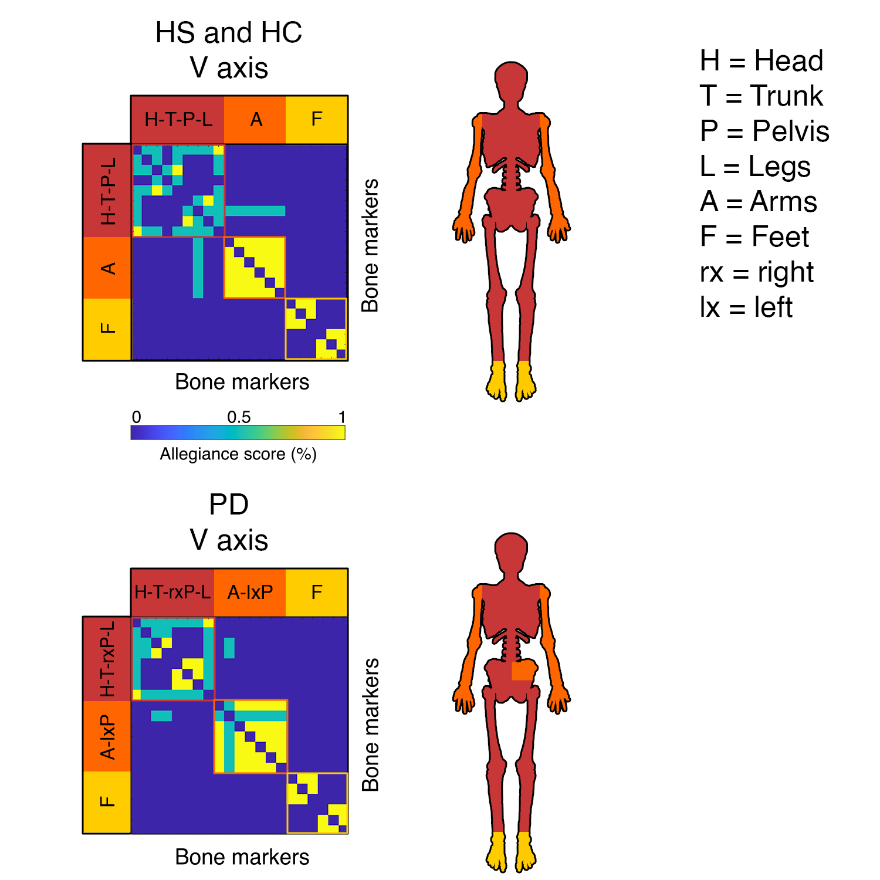


**Figure S5. Kinematic modular organization of the kinectomes on the vertical axis**

Allegiance matrices for cluster analysis, based on the Louvain method and consensus-clustered through 100 iterations. The algorithm automatically defines which body parts belong to the same community, suggesting a functional relationship among those elements. Each matrix includes clustering information from both accelerations and jerks. Healthy subjects (HS) and healthy controls (HC) share the same communities. Parkinson’s disease group (PD) shows very similar organization, except for the left side of the pelvis. Body parts depicted with the same color belong to the same functional community.

**
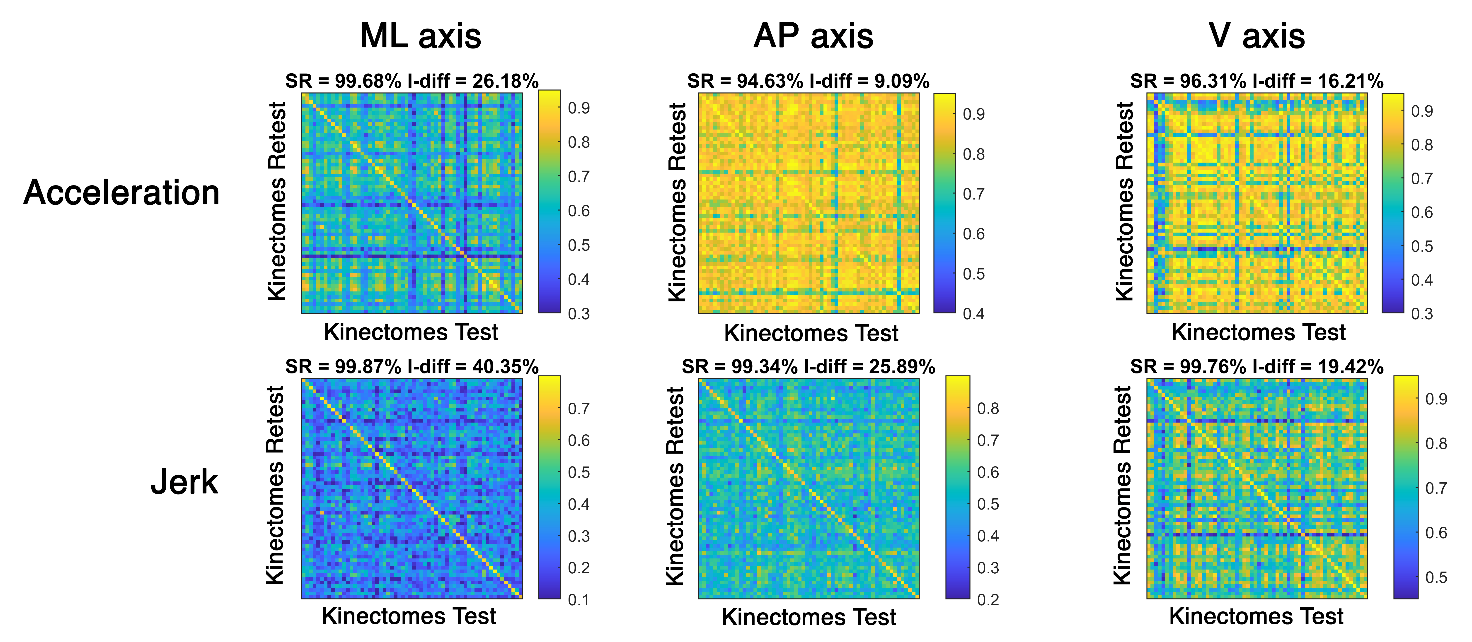
**

**Figure S6. Identifiability matrices for fingerprinting in the healthy group**

Identifiability matrices of the healthy subjects (HS) for the acceleration and jerk parameters in mediolateral (ML), anteroposterior (AP), and vertical (V) axes. On top of each matrix the identification rate (IR) and the differential identifiability (I-diff) are reported. The identifiability matrices of the HS groups (60 subjects) performed similarly to the healthy controls (23 subjects).


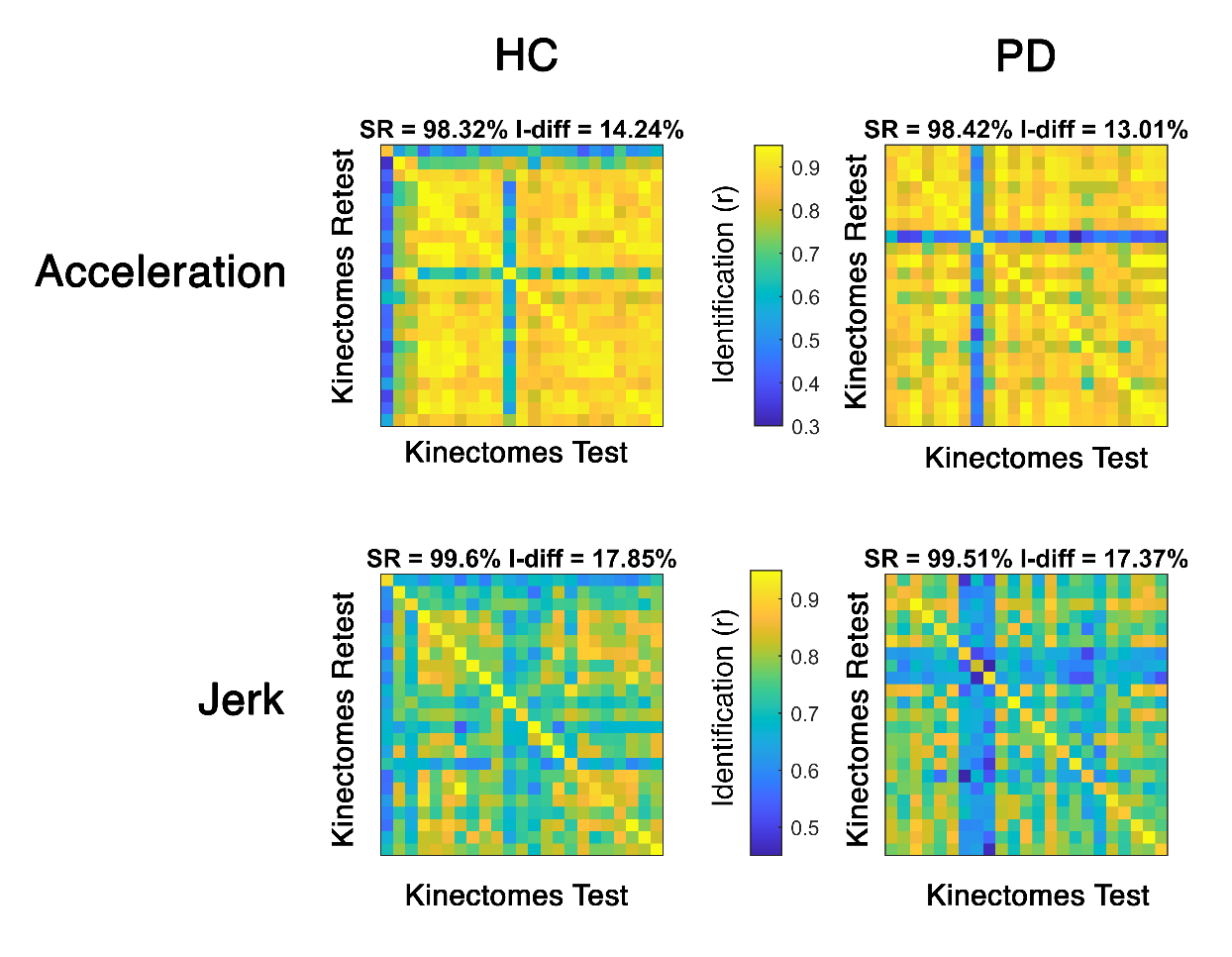


**Figure S7. Identifiability matrices of the vertical kinectomes in patients and controls**

Identifiability matrices of the patients with Parkinsons’ disease group (PD) and healthy controls (HC) for the acceleration and jerk parameters in the vertical (V) axis. On top of each matrix the identification rate (IR) and the differential identifiability (I-diff) are reported.


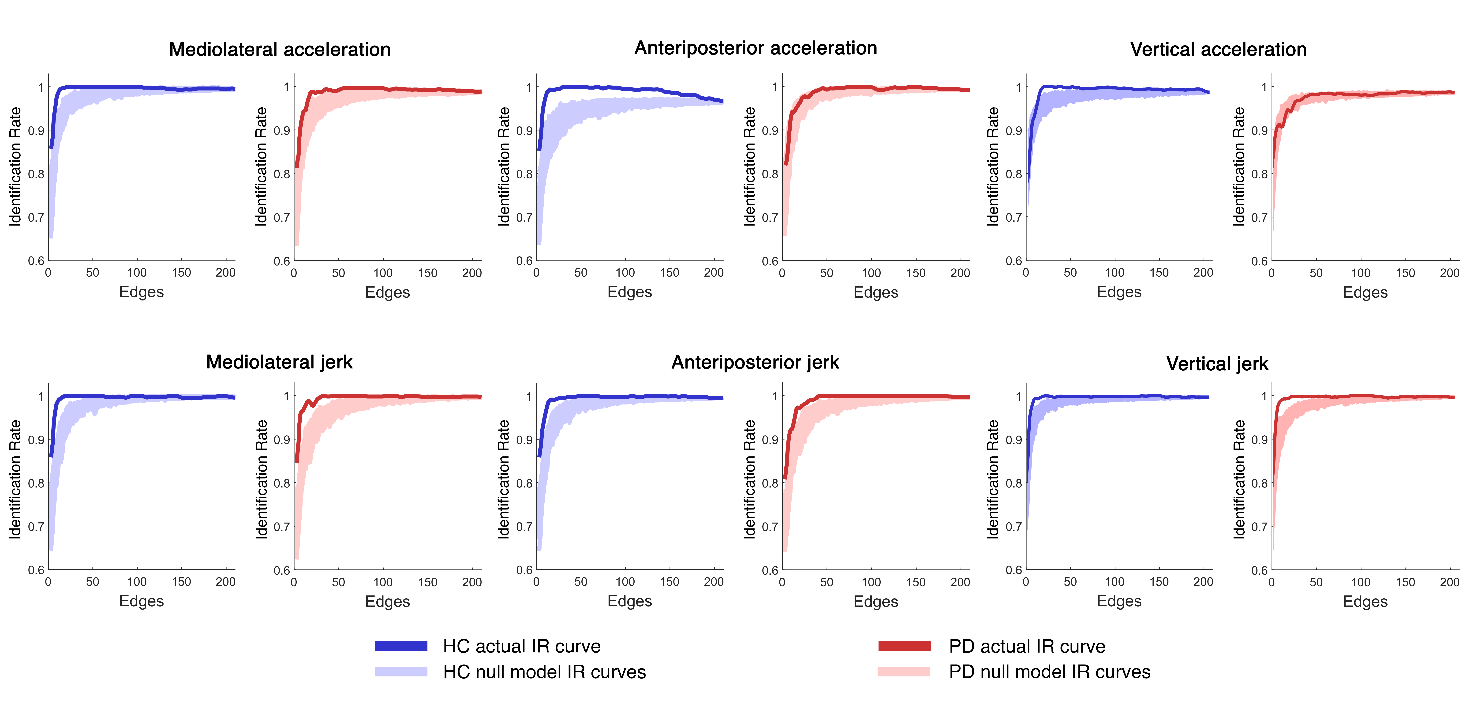


**Figure S8. Identification rate with null models**

Identification rate (IR) for the kinectomes of healthy controls (HC) and patients (PD), for acceleration and jerk in mediolateral (ML), anteroposterior (AP) and vertical (V) axes, with the corresponding null model IR curves. The IR is computed iteratively: starting from three edges, at each iteration one edge is added and the IR is computed. The actual IR curves are built including the edges in order based on their contribution to identifiability (from the most to the least contributing), according to the intraclass correlation (ICC) analysis. The null model IR curves are built the same way as the actual IR curves, but adding random edges (as opposed to adding first the one contributing the most to identifiability) in a random order. Noteworthy, the actual curves (i.e., with ICC-ordered edges) performed better than the null model curves (i.e., with randomly ordered edges).
